# A Strategy for the Rapid Development of a Safe *Vibrio cholerae* Candidate Vaccine Strain

**DOI:** 10.3390/ijms222111657

**Published:** 2021-10-28

**Authors:** Dmitry S. Karpov, Anna V. Goncharenko, Evgenii V. Usachev, Daria V. Vasina, Elizaveta V. Divisenko, Yaroslava M. Chalenko, Andrei A. Pochtovyi, Roman S. Ovchinnikov, Valentin V. Makarov, Sergei M. Yudin, Artem P. Tkachuk, Vladimir A. Gushchin

**Affiliations:** 1 Center for Precision Genome Editing and Genetic Technologies for Biomedicine, Engelhardt Institute of Molecular Biology, Russian Academy of Sciences, Vavilov str., 32, 119991 Moscow, Russia; 2 Bach Institute of Biochemistry, Federal Research Centre “Fundamentals of Biotechnology” of the Russian Academy of Sciences, 119071 Moscow, Russia; pylaevanna@gmail.com (A.V.G.); evgenyvusachev@gmail.com (E.V.U.); d.v.vasina@gmail.com (D.V.V.); artem.p.tkachuk@gmail.com (A.P.T.); wowaniada@gmail.com (V.A.G.); 3 N.F. Gamaleya Federal Research Centre for Epidemiology and Microbiology, Ministry of Health of the Russian Federation, Gamaleya str., 18, 123098 Moscow, Russia; elizaveta.divisenko@yandex.ru (E.V.D.); yaroslavazaka@yandex.ru (Y.M.C.); a.pochtovyy@gmail.com (A.A.P.); rsovchinnikov@mail.ru (R.S.O.); 4 Department of Virology, Biological Faculty, Lomonosov Moscow State University, 119991 Moscow, Russia; 5 Centre for Strategic Planning of FMBA of Russia, 119121 Moscow, Russia; makarov@cspmz.ru (V.V.M.); yudin@cspmz.ru (S.M.Y.)

**Keywords:** *Vibrio cholerae*, genome engineering, synthetic reporter operon, amilCP, candidate vaccine strain

## Abstract

Approximately 1/6 of humanity is at high risk of experiencing cholera epidemics. The development of effective and safe vaccines against *Vibrio cholerae,* the primary cause of cholera, is part of the public health measures to prevent cholera epidemics. Natural nontoxigenic *V. cholerae* isolates represent a source of new genetically improved and relatively safe vaccine strains. However, the genomic engineering of wild-type *V. cholerae* strains is difficult, and these strains are genetically unstable due to their high homologous recombination activity. We comprehensively characterized two *V. cholerae* isolates using genome sequencing, bioinformatic analysis, and microscopic, physiological, and biochemical tests. Genetic constructs were Gibson assembled and electrotransformed into *V. cholerae*. Bacterial colonies were assessed using standard microbiological and immunological techniques. As a result, we created a synthetic chromoprotein-expressing reporter operon. This operon was used to improve the *V. cholerae* genome engineering approach and monitor the stability of the genetic constructs. Finally, we created a stable candidate *V. cholerae* vaccine strain bearing a *recA* deletion and expressing the β-subunit of cholera toxin. Thus, we developed a strategy for the rapid creation of genetically stable and relatively safe candidate vaccine strains. This strategy can be applied not only to *V. cholerae* but also to other important human bacterial pathogens.

## 1. Introduction

Pathogenic strains of *Vibrio cholerae* cause cholera, an acute infectious disease characterized by a sharp loss of water due to profuse diarrhoea, which can be fatal in the absence of rehydration therapy [1]. It is estimated that approximately 1.3 billion people are at high risk in endemic cholera countries [2]. The periodically occurring cholera epidemics and their local outbreaks in Africa, South Asia, and Southeast Asia are of great concern. Moreover, the appearance of multidrug-resistant *V. cholerae* strains replacing antibiotic-susceptible strains [3] increases the risk of high severity of future cholera epidemics and outbreaks. Therefore, *V. cholerae* represents a global health threat, stimulating the development of public health measures to limit the spread of pathogenic strains to prevent cholera epidemics. Long-term efforts to improve water quality, sanitation, and hygiene are not enough for successful cholera control in developing countries. Vaccination represents a highly effective short- or medium-term strategy for cholera control. Currently, vaccination with oral cholera vaccines (OCVs) is considered by the World Health Organization to be an essential tool in cholera outbreak prevention and control. However, clinical studies have revealed lower protectiveness of OCVs in children <5 years of age compared with older individuals [4]. Moreover, live attenuated *V. cholerae* strains show better protection and efficacy [5,6,7]. In addition, two- or higher-dose vaccine regimens, although more effective, are more expensive and less feasible than a single-dose regimen. These data indicate the need for further improvement of cholera vaccines.

Genetic engineering represents one of the ways to improve cholera vaccine strains. Genetically engineered vaccine strains can be divided into two groups. The first group comprises attenuated pathogenic strains that lack toxic genes. The second group comprises natural nontoxigenic strains expressing immunogenic but nonactive toxic proteins. The genetically attenuated strain HaitiV is an example of the first group [8]. This strain was obtained by rational design of the pathogenic strain of the *V. cholerae* serovar El Tor, which caused an outbreak in 2010 in Haiti [9]. VA1.3 is an example of the second group and was constructed from the nontoxigenic strain of *V. cholerae* El Tor, Inaba, by integrating multiple copies of *ctxB* into the *hly* locus [10]. Natural nontoxigenic strains have advantages over attenuated pathogenic strains because they are less toxic, require less genetic modification, and usually have active defence systems that protect them from infection by toxigenic phages such as CTX. However, *Vibrio* strains can be poorly transformable or nontransformable [11] due to the presence of DNases encoded in the genome or by mobile genetic elements [12,13,14]. *V. cholerae* strains also have active clustered regularly interspaced short palindromic repeats (CRISPR)/Cas systems [15,16] that can also interfere with bacterial transformation [17,18,19].

Here, we developed a strategy for fast engineering of a new *V. cholerae* candidate vaccine strain using a chromoprotein-based synthetic reporter operon. The strategy consists of the four steps: (1) characterization of natural bacterial isolates to choose the candidate; (2) optimization of the bacterial cell transformation protocol; (3) development of an amilCP-expressing reporter operon; and (4) genome integration of the reporter operon expressing an immuno-stimulating protein with simultaneous deletion of the *recA* locus.

## 2. Results

### 2.1. Identification and Initial Characterization of Natural V. cholerae Isolates

In the first step of our strategy for creating a candidate vaccine strain, we used several methods to identify and characterize two natural nontoxigenic *V. cholerae* strains, designated strains 31 and 41. According to the MALDI-TOF mass spectrometry results obtained with a MALDI BioTyper, strains 31 and 41 were identified as *Vibrio albensis* (with identification scores equal to 2.01 and 1.88, respectively) (Figure S1). *V. albensis* is the name for species comprising non-O1 and non-O139 *V. cholerae* strains [20,21]. However, the identification scores allow us only to reliably conclude that both strains belong to the *Vibrio* genus. Although microscopic examination and sugar utilization tests also indicated that our strains have *Vibrio*-like features (Figures S2 and S3), these results were not enough to identify the strains. To further increase the identification reliability, we sequenced the genomes of our strains on the Ion S5XL platform (Thermo Scientific, Waltham, MA, USA). 16S RNA sequences extracted from the assembled genomes suggested that both strains belonged to the genus *Vibrio*. Next, a genome-based identification of strains was performed using the maximum average nucleotide identity (ANI) obtained from paired comparisons with all the non-redundant genomes available in the MIGA and the NCBI Genome databases using the NCBI Prok tool on the Microbial Genomes Atlas web server [22]. The genomes of strains 31 and 41 were most similar to those of *V. cholerae* LMA3984 (ANI 98.95%) and *V. cholerae* NZ (ANI 98.77%), respectively. Then, an MLST analysis performed with the MLST 2.0 web server showed that both strains belong to *V. cholerae*. Strain 31 has the sequence type 760, while strain 41 has a new combination of alleles, and the nearest sequence type is 1317. Thus, genetic analysis strongly suggested that our strains belong to the species *V. cholerae*.

Next, draft genome sequences were used to search for the presence of CRISPR/Cas systems using CRISPRminer [23]. We found CRISPR/Cas components of type IF systems with spacers against various *V. cholerae* phages in both strains. Consistently, only fragments of prophages (Table S3) were present in the strains’ genomes. Moreover, as indicated in the strains’ passports, they are resistant to various phages. Therefore, the data suggest that the identified CRISPR/Cas systems are active.

Then, we searched for virulence genes and genes that provide resistance to known antibiotics. In the case of strain 31, 148 factors with varying degrees of integrity of the reading frame were found. In the case of strain 41, 140 factors were found. According to the BLAST search results, the strains have some genes encoding accessory toxins (Table S3). Most of these genes are inactivated by indels, while some of them seem to be active. Therefore, the ORF of the *hlyA* gene encoding haemolysin in strain 41 has no errors, which is consistent with the formation of large α-haemolysis zones (Figure S4). In contrast, the *hlyA* gene in strain 31 was inactivated by indels, and accordingly, the colonies formed weak β-haemolysis zones (Figure S4). We concluded that strain 31 is safer and used it in subsequent experiments.

### 2.2. Optimization of V. cholerae Transformation

**Figure 1:**
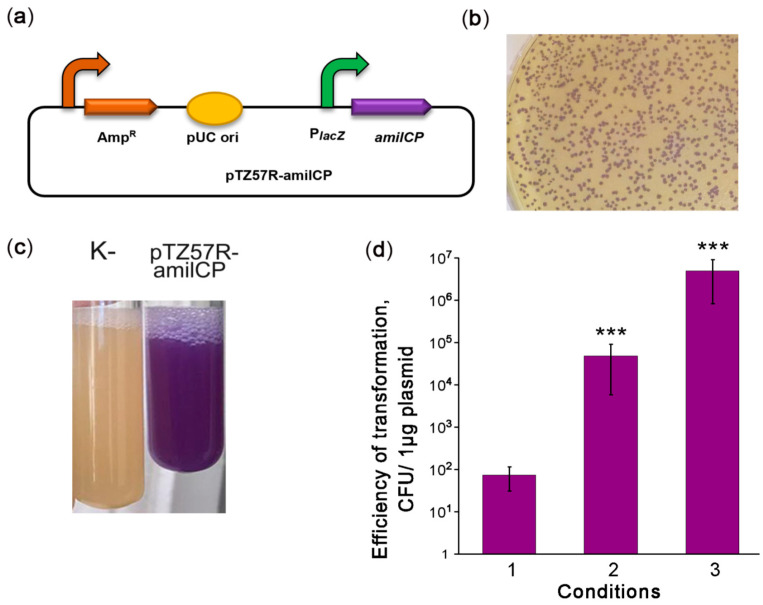
Optimization of *V. cholerae* electrotransformation. (**a**) Scheme of the episomal reporter plasmid pTZ57R-amilCP. PlacZ: promoter of the LacZ α-peptide, amilCP: the gene encoding the purple chromoprotein. The curved arrows denote promoters; (**b**) colonies of *V. cholerae* transformed with pTZ57R-amilCP; (**c**) stationary-phase liquid cultures of *V. cholerae* strains that were nontransformed (K-) or transformed with the plasmid pTZ57R-amilCP; and (**d**) improvement of the protocol for *V. cholerae* transformation by electroporation. CFU: colony-forming unit. The conditions for transformation are described in the text. The data are presented as the mean (*n* = 3) ± SD. Statistical significance was calculated using Student’s two-tailed *t*-test for comparing two independent means. *** indicates *p* < 0.001.

Highly efficient transformation is essential for effective genetic engineering of a chosen bacterial strain. In the second step of our strategy, we optimized the transformation of *V. cholerae* strain 31. Therefore, we constructed the reporter plasmid pTZ57R-amilCP encoding the amilCP purple chromoprotein (CP) and having an *E. coli* pUC origin (Figure 1a) that can be maintained in *Vibrio* species [12,24]. Using the protocol for *V. cholerae* transformation by electroporation as described in [25], we obtained 10^1^–10^2^ CFU per 1 μg of plasmid (Figure 1b, condition 1). However, this transformation efficiency was not enough to modify the *V. cholerae* genome with an integrative construct. We hypothesize that the low transformation efficiency may be caused by DNA degradation. In the protocol [25], the cells are used in the logarithmic growth phase, when *V. cholerae* secretes two extracellular DNases, Dns and Xds. Dns endonuclease is responsible for plasmid degradation [26], while Xds endonuclease is responsible for the degradation of linear DNA [27]. The genome of the *V. cholerae* 31 strain contains complete sequences for genes encoding both DNases. Dns is repressed in the stationary phase by a quorum sensing-dependent mechanism [26,28]. Therefore, we used a stationary phase culture for transformation. This allowed us to increase the transformation efficiency significantly to approximately 10^5^–10^6^ CFU per 1 μg of the plasmid (Figure 1b, condition 2). Using cells grown to the stationary phase on minimal M9 medium further increased the efficiency of *V. cholerae* transformation to 10^6^–10^7^ CFU per 1 μg of plasmid (Figure 1b condition 3). Thus, we optimized the conditions for the high-efficiency transformation of natural *V. cholerae* strains by electroporation.

### 2.3. Optimization of Synthetic Reporter Operon Expression

**Figure 2:**
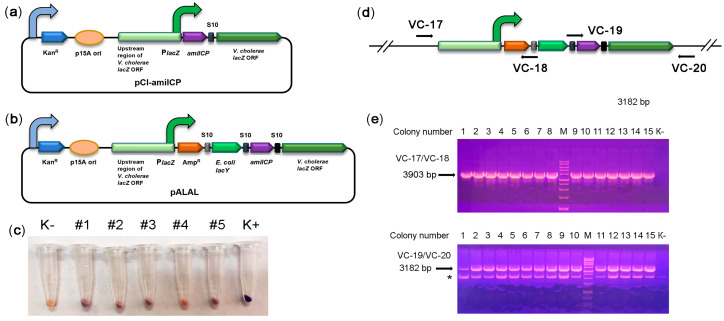
Integration of the synthetic reporter operon into the *lacZ* locus. (**a**) Schematic depiction of the integration reporter plasmid pCI-amilCP. The curved arrows denote promoters; (**b**) schematic depiction of the integrative pALAL plasmid carrying the synthetic reporter operon containing the genes for β-lactamase (AmpR), *E. coli* galactopermease (*lacY*), chromoprotein (amilCP), and *V. cholerae* β-galactosidase (*lacZ*); (**c**) colour of cell pellets of *V. cholerae* transformed with the integrative plasmid pALAL. An untransformed *V. cholerae* culture (K-) was used as a negative control. *V. cholerae* transformed with the plasmid pTZ57R-amilCP was used as a positive control. (**d**) Design of PCR to check the correct integration of the reporter operon. The positions of primers are marked with arrows. If the construct is integrated at the correct genomic position, the primer pair VC-17/VC-18 should yield a 3903 bp fragment, and the VC-19/VC-20 pair should yield a 3182 bp fragment; (**e**) PCR analysis of the edited *V. cholerae* colonies. PCR fragments were separated in a 1.5% agarose gel in the presence of EtBr. Genomic DNA from nontransformed strain 31 was used as a negative control (K-). M: 1 kb DNA marker, 1–15: colonies used in the analysis. An asterisk (*) indicates the non-specific band.

In the third step, we optimized the expression of the amilCP-based reporter operon. The promoter dramatically affects heterologous protein expression in *V. cholerae* both in vitro and in vivo [29,30,31]. Previously, the *V. cholerae lacZ* locus was used as a safe site for integrating genetic constructs [32,33], and the *lacZ* promoter was utilized to express heterologous proteins [34]. Therefore, we created the integration construct pCI-amilCP for insertion of amilCP into the *lacZ* locus (Figure 2a). This construct harboured the amilCP-lacZ synthetic operon under the control of the natural *lacZ* promoter. Integration flanks were obtained from the other *V. cholerae* strain, P-19241. Since they differ in sequence from the *V. cholerae* strain 31 *lacZ* locus, we identified the part of the *lacZ* locus that came from the integration plasmid. The integration flanks were 3 kb long; flanks of such length provide high integration efficiency [35]. After pCI-amilCP transformation, none of the grown colonies had colour imparted by amilCP. We hypothesized that the amilCP expression level from the integrated construct was not sufficient to colourize the colonies.

Next, we tried to use *lacZ* as a reporter gene. The genome of strain 31 harbours a complete β-galactosidase gene without deleterious mutations. Accordingly, the ONPG test confirmed the presence of β-galactosidase activity in the cell lysates (Figure S5). As expected for *V. cholerae* strains, the genome of our strain has no gene for high-affinity 5-bromo-4-chloro-3-indoyl-beta-D-galactopyranoside (X-Gal) transport, such as LacY galactopermease. Unexpectedly, strain 31 was able to utilize X-Gal (Figure S6). The colonies developed colour slowly within 48 h, suggesting the presence of some low-activity transporters for X-Gal. The possible transporters for X-Gal can be ABC transporters encoded by the *mgl* operon and involved in methylgalactoside and galactose transport in gram-negative bacteria [36,37]. Previously, the *E. coli lacYZ* operon was used as a strong reporter for in vivo identification of infection-inducing genes in pathogenic *V. cholerae* strains [38]. Therefore, we took advantage of active β-galactosidase and added the *E. coli lacY* gene to the synthetic reporter construct (Figure 2b). Genes were separated by spacers from the *V. cholerae* S10 operon of the ribosomal proteins. We reasoned that S10 spacers should provide a high level of mRNA translation. We expected that colonies expressing the improved reporter operon would develop colour more rapidly. Indeed, in *E. coli*, the *lacY-*containing synthetic reporter causes *E. coli* colonies to develop more intense colouration (Figure S7). However, the improved reporter operon did not enhance *V. cholerae* transformant colouration intensity on X-Gal plates (data not shown). We hypothesize that X-Gal transport is not the rate-limiting process of colour development in our strain.

To grow only transformed colonies, we added the β-lactamase gene into the synthetic reporter operon, yielding the pALAL integrative plasmid (Figure 2b). The β-lactamase gene should mediate resistance to ampicillin in transformed cells that are otherwise sensitive to it (Table S4). *V. cholerae* transformation with the pALAL plasmid provided 10^1^–10^2^ colonies per 1 μg of the plasmid on ampicillin-supplemented agar plates. The cell pellets of several randomly picked transformed colonies 2, 3, and 5 clearly had a darker colour than control non-transformed cells, suggesting a low level of amilCP accumulation (Figure 2c). PCR with pairs of the primers VC-17/VC-18 and VC-19/VC-20 verified the integration of the reporter operon into the target *lacZ* locus in these and other recombinant *V. cholerae* colonies (Figure 2d,e). Sequencing of PCR products further confirmed the correct integration of constructs.

Moreover, we also tried to transform *V. cholerae* with linear DNA. Therefore, we used a 9082 bp integration cassette amplified from the pALAL plasmid with the primers VC05 and VC09. As a result, we obtained only one ampicillin-resistant colony (# 1). This colony had no purple colour. However, the cell pellet from this colony had a darker colour than that in the negative control (Figure 2c), suggesting a low level of amilCP accumulation. PCR confirmed correct integration of the construct in the *lacZ* locus (Figure 2e). Unexpectedly, sequencing revealed the integration of a 516 bp fragment of the p15A plasmid ori next to the left flank of the integrated construct. We hypothesize that the PCR fragment was destroyed upon transformation (apparently, by the Xds exonuclease). Seemly, the pALAL plasmid copurified with the PCR product was integrated into the genome instead of the PCR product. Therefore, we may conclude that our strain can be transformed only by circular plasmids.

**Figure 3:**
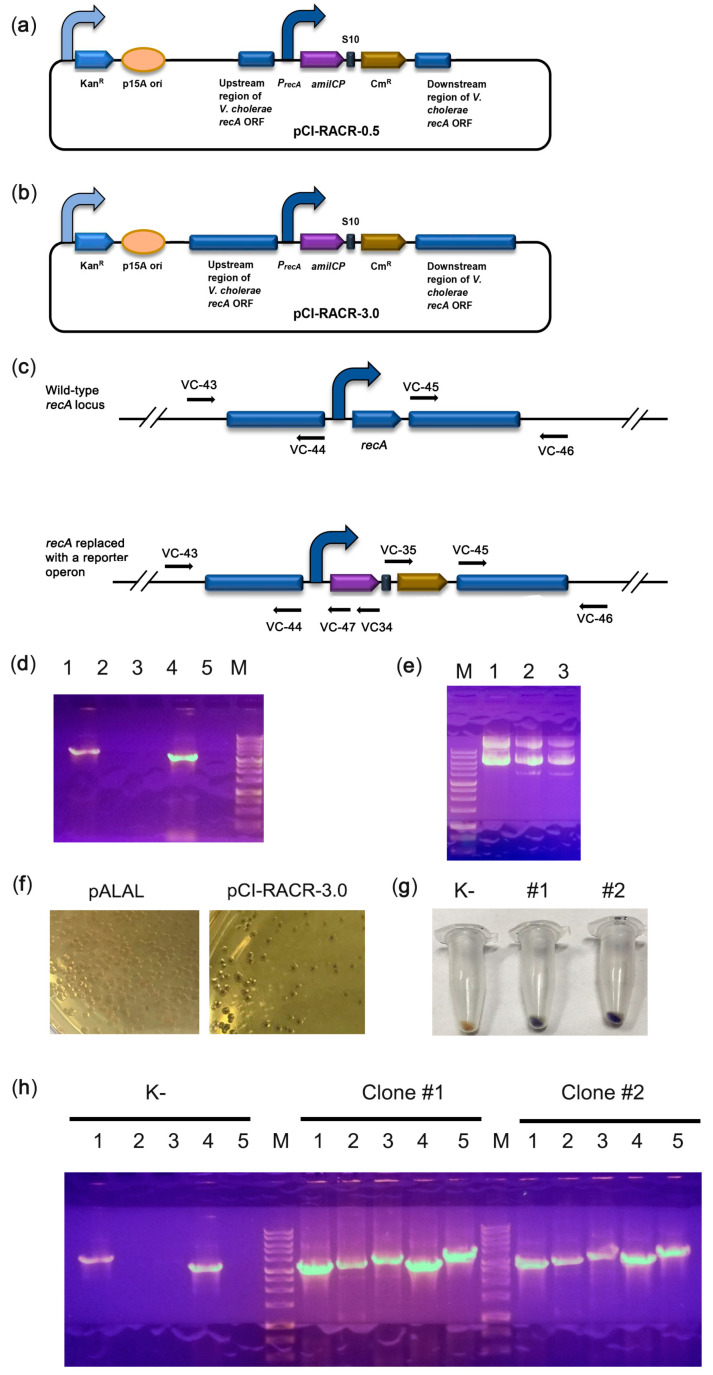
Integration of the synthetic reporter operon into the *recA* locus. (**a**,**b**) Schematic depiction of the pCI-RACR reporter constructs. The curved arrows denote promoters; (**c**) scheme of the experiment for PCR verification of construct integration. The positions of primers are marked with arrows; (**d**) PCR check for the integration of pCI-RACR-0.5. If the construct was integrated into the correct genomic position, the VC-43/VC-44 pair amplified a fragment of 3157 bp (lane 1), the VC-43/VC-47 pair amplified a fragment of 3330 bp (lane 2), the VC-43/VC-34 pair amplified a fragment of 3824 bp (lane 3), the VC-45/VC-46 pair amplified a fragment of 3250 bp (lane 4), and the VC-35/VC-46 pair amplified a fragment of 3929 bp (lane 5). (**e**) Plasmid preparations from *E. coli* (lane 1) or *V. cholerae* colonies transformed with pCI-RACR-0.5 (lanes 2 and 3); (**f**) colour of *V. cholerae* colonies transformed with the pALAL or pCI-RACR-3.0 integrative plasmid; (**g**) colour of cell pellets of *V. cholerae* transformed with the integrative pCI-CR-3.0. An untransformed *V.* *cholerae* culture (K-) was used as a negative control; (**h**) PCR check for the integration of pCI-RACR-3.0. Untransformed *V. cholerae* strain 31 (K-) was used as a negative control. The primers used were the same as those described in part (**c**) of the figure.

Our results suggest that the native *lacZ* promoter is relatively weak and cannot provide sufficient expression levels of reporter proteins to visualize transformed colonies. Therefore, we used the stronger promoter of *recA*, which highly and stably expresses the housekeeping gene in *V. cholerae* [31]. We assembled two plasmids bearing a synthetic operon consisting of the amilCP and chloramphenicol acetyltransferase genes under the control of the *recA* promoter and terminator regions. The plasmid pCI-RACR-0.5 bears short *recA* flanks that are 0.5 kb long (Figure 3a) and is supposed to be episomal, while the pCI-RACR-3.0 plasmid bears 3 kb-long flanks (Figure 3b) and is expected to be integrated into the *recA* locus. As expected, pCI-RACR-0.5 was not integrated into the *recA* locus. This was confirmed by PCR (Figure 3c,d) and the presence of the episomal plasmid in cell cultures (Figure 3e). The *V. cholerae* colonies, as in the case of pTZ57R-amilCP, were coloured purple. *V. cholerae* colonies transformed with pCI-RACR-3.0 exhibited a purple centre (Figure 3f), and their pellets were also clearly purple in colour (Figure 3g). PCR confirmed the correct integration of pCI-RACR-3.0 into the *recA* locus (Figure 3h). Next, we found that *V. cholerae* colonies grown on brain heart infusion (BHI) medium for 72 h developed a more intense colour (Figure S8). Therefore, we optimized the expression of the synthetic operon to identify transformed *V. cholerae* colonies simply by visual inspection.

### 2.4. Assessing the Stability of the Genetic Constructs

**Figure 4:**
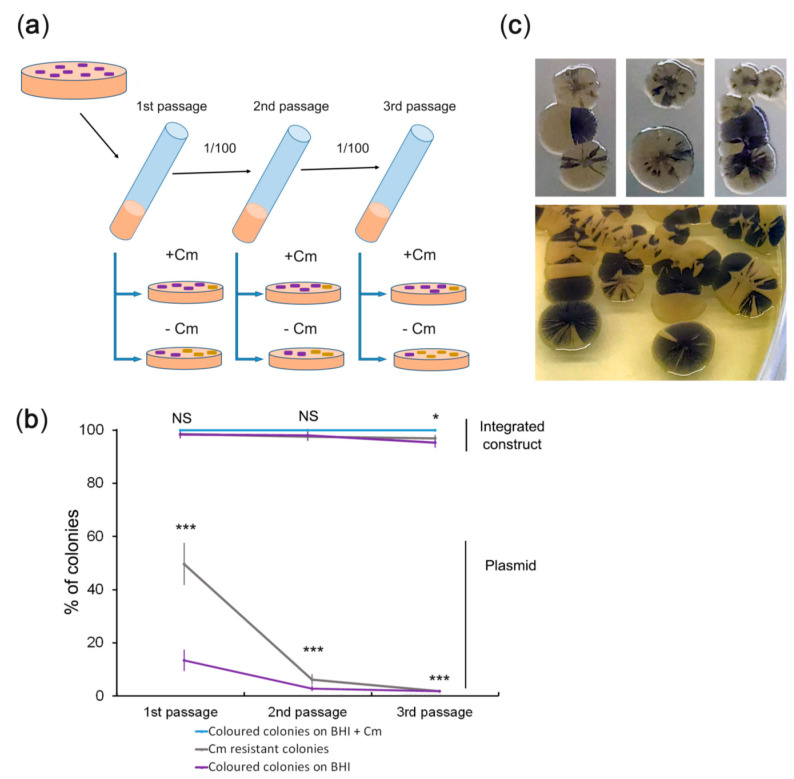
A test for stability of the genetic constructs. (**a**) Scheme of the experiment for assessing the stability of the genetic constructs. The chosen colonies were grown in BHI medium without antibiotics for 16 h at RT. Equal volumes of cultures of the same passages were diluted 10^6^- or 10^7^-fold, plated on BHI plates with or without Cm, and incubated at RT for 72 h. One-third of the first overnight culture was transferred to a tube with fresh BHI medium without antibiotics and grown for 24 h at RT. Then, the plating of the grown cultures was repeated. (**b**) Quantitative data on the genetic construct stability test. *V. cholerae* cultures grown to the stationary phase were diluted 10^6^-fold (1st and 3rd passages) or 10^7^-fold (2nd passage), plated, and grown on BHI agar plates. Representative photos of the plates are shown in Figure S9. Colonies were counted using a Clono Counter [39] or manually depending on the number of colonies to be counted. The number of coloured colonies grown in the presence of Cm was set to 100%. The data are presented as the mean (*n* = 5) ± SD. Statistical significance: NS: nonsignificant differences, * *p* indicates between 0.05 and 0.01, *** indicates *p* < 0.001 according to one-way ANOVA test; (**c**) colour heterogeneity of *V. cholerae* colonies transformed with the plasmid. After the first passage, *V. cholerae* cultures were diluted 10^6^-fold, spread on BHI agar plates without antibiotics, and incubated for 72 h at RT.

Colony colouration due to the expression of amilCP as a reporter protein is the fastest and easiest way to monitor the presence of genetic constructs and track their stability. To this end, we used episomal pCI-RACR-0.5 and integrative pCI-RACR-3.0 plasmids. The scheme for the stability test is presented in Figure 4a. According to the stability test results (Figure 4b and Figure S9), the colonies with the integrated construct displayed only a subtle decrease in the percentage of coloured and Cm-resistant colonies: 93.6 and 95.2% of the corresponding colonies were observed after the 3rd passage. Simultaneously, the colonies transformed with the episomal plasmid showed a dramatic decrease in coloured and Cm-resistant colonies: 1.3% of coloured colonies were observed after the 3rd passage. Generally, the number of antibiotic-resistant colonies was higher than the number of coloured colonies. This was more obvious for colonies transformed with the episomal plasmid. We hypothesize that the copy number of genes producing an enzyme can be as low as one or two to provide cellular resistance to antibiotics. In contrast, the copy number should be significantly high to provide enough CP expression to colourize the cells. We observed the same situation for the integrative construct when the copy number was not enough to colourize the colonies but enough to provide ampicillin resistance. We then observed the sectoral colouring (Figure 4c). The colourless sectors seemingly appeared due to plasmid loss or a rapid decrease in the plasmid copy number. These data suggest that the integrated construct was highly stable, while the plasmid was rapidly lost. Notably, in the presence of Cm, only purple colonies grew, suggesting that both genetic constructs were stable under antibiotic selection.

### 2.5. Construction of a Candidate V. cholerae Vaccine Strain

**Figure 5:**
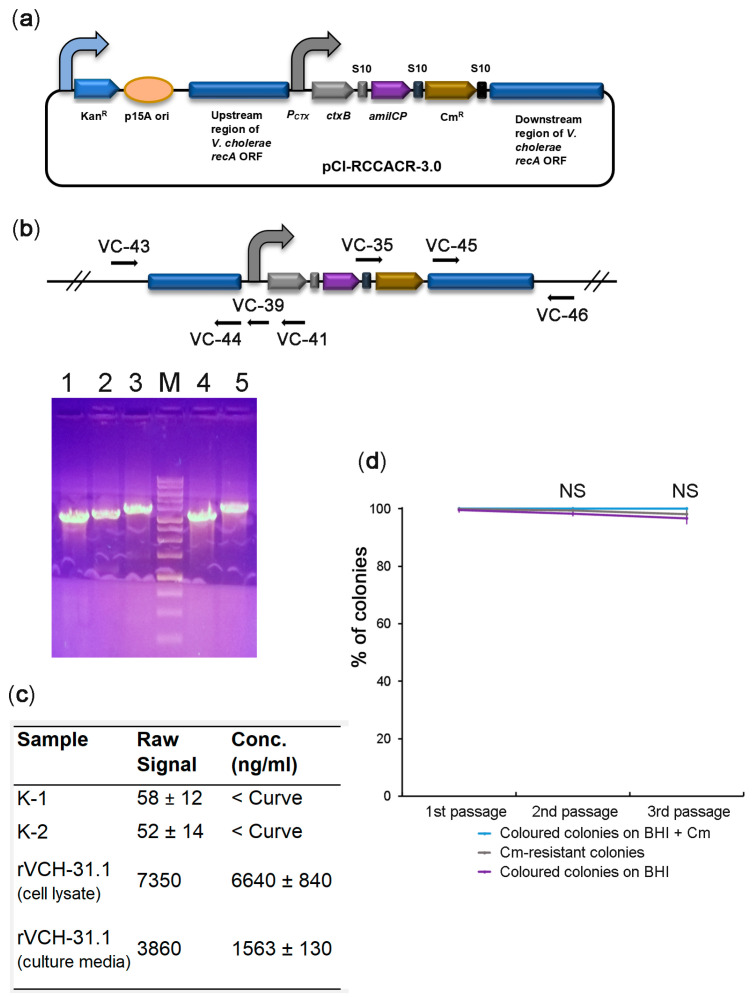
Construction of a candidate *V. cholerae* vaccine strain expressing cholera toxin β-subunit. (**a**) Schematic depiction of the pCI-RCCACR-3.0 integrative construct bearing a synthetic operon with *ctxB*, the *amilCP* reporter, and the *cat* marker genes. The curved arrows denote promoters; (**b**) PCR check for the integration of pCI-RCCACR-3.0. The positions of the primers are marked with arrows. In the case of correct genomic integration of the construct, the VC-43/VC-44 primer pair amplified a fragment of 3157 bp (lane 1), the VC-43/VC-39 primer pair amplified a fragment of 3368 bp (lane 2), the VC-43/VC-41 primer pair amplified a fragment of 3759 bp (lane 3), the VC-45/VC-46 primer pair amplified a fragment of 3250 bp (lane 4), and the VC-35/VC-46 primer pair amplified a fragment of 3929 bp (lane 5). M: DNA molecular weight marker; (**c**) results of GM1-ELISA of *ctxB* production by the rVCH-31.1 strain. The data are presented as the mean (*n* = 3) ± SD. The negative controls were the original *V. cholerae* nontoxigenic strain (K-1) or recombinant strain edited with the pCI-RACR-3.0 plasmid (K-2). ‘<Curve’ indicates that the signal was below the calibration curve (Figure S10); (**d**) quality control of the *V. cholerae* candidate vaccine strain by the genetic stability test. The data are presented as the mean (*n* = 5) ± SD. NS: nonsignificant differences according to one-way ANOVA.

In the final step, we proceeded to construct a candidate vaccine strain. Since the cholera toxin β-subunit (CtxB) is safe and provides protective properties for vaccines, e.g., for the widely used rBS-WC (Dukoral) [40,41,42], we sought to convert our strain to a CtxB producer. To achieve this goal, we assembled constructs to integrate a synthetic operon consisting of *ctxB*, *amilCP*, and *cat* into the *recA* locus. However, in all the constructs purified from violet *E. coli* colonies resistant to Cm, the *ctxB* ORF was damaged, leading to inactive protein synthesis. We hypothesize that high levels of the cholera toxin produced from the strong constitutive promoter are highly toxic to *E. coli*. Therefore, the surviving colonies harboured the assembled constructs with *ctxB* mutations. We reasoned that to assemble the construct correctly, we needed to use a promoter that is weak or inactive in *E. coli* and active in *V. cholerae*. The promoter of the cholera toxin operon is very weak in *E. coli* [43]. At the same time, it is highly active in *V. cholerae* and directly regulated by two transcription factors, toxT [44] and toxR [45]. Indeed, the construct (Figure 5a) was assembled in *E. coli* correctly, suggesting that the β-subunit was expressed at nontoxic levels. The obtained integration construct was transformed into *V. cholerae*. The colony with the most intense violet colouration was further analysed. PCR confirmed the correct integration of the *ctxB*-containing operon into the *recA* locus (Figure 5b). The recombinant strain produced the cholera toxin β-subunit at 6.64 µg/mL in the cell lysate and about 1.6 µg/mL in the culture media (Figure 5c). The productivity of our strain is close to one of the best CTB-producing V. cholerae recombinant strains M7922-C1, which produces β-subunit at 3.17 ± 1.69 µg/mL [46]. The genetic stability test suggests the high stability of the integrated *ctxB*-expressing operon (Figure 5d). Thus, we constructed a novel *V. cholerae* candidate vaccine strain designated rVCH-31.1 suitable for further preclinical studies.

## 3. Discussion

Here, we provide a four-step strategy for using an amilCP-based reporter system for the rapid development and quality control assessment of candidate vaccine strains against important human pathogens using *V.* *cholerae* as an example.

In the first step of our strategy, genomic, bioinformatics, biochemical, and physiological data should be used to select the safest strain from natural isolates as a candidate for further genetically engineered strains. One of the criteria for candidate strain safety is the absence of highly active toxic genes. For example, because strain 31 has the inactive *hlyA* gene (Figure S3), it is safer than strain 41. At the same time, both strains have other intact genes related to *V. cholerae* pathogenicity. One of these genes, *hap* encodes haemagglutinin protease. Others belong to the *rtx* operon: *rtxC* encodes the activator for the *rtxA* toxin, and *rtxB* encodes for the ABC transporter of the *rtxA* toxin. However, the *rtxA* encoding toxin is inactivated by indels in both strains, so the *rtx* system, seemly, is not pathogenic. Therefore, if left intact, hap can contribute to potential adverse properties for future live cholera vaccines. On the other hand, the vaccine strain having some weak virulent factors should stimulate the development of a more comprehensive immune response inactivating several pathogenic factors and thereby having higher protective potential against cholerae than the entirely non-toxic strain.

The second step of the strategy is to develop a protocol for high-efficiency transformation of a chosen bacterial strain. Effective genome engineering of *V. cholerae* is impossible without high-efficiency transformation, which allows us to make several genome changes, e.g., mutation or deletion of several genes encoding toxins, to increase the vaccine strain’s safety. The high transformation efficiency of *V. cholerae* strains can be achieved by inducing their natural competence in the presence of chitin [47]. We also achieved high transformation efficiency through electroporation after incubating cells on M9 minimal medium to the stationary phase. In the stationary phase, upon activation of quorum sensing, the expression of *dns* encoding highly active extracellular DNase is inhibited in a HapR-dependent manner [28,31]. However, the other highly active extracellular exonuclease, Xds, is not controlled by cell density and remains active in stationary-phase cells [48]. This explains the inability of linear DNA fragments to transform our *V. cholerae* strain under the conditions we used. At the same time, cells in the natural competence state can be transformed with linear DNA [35]. Therefore, we speculate that the incubation of *V. cholerae* on a minimal medium without the addition of chitin might induce a high-competence state distinct from chitin-induced competence.

The third step of the strategy is the creation of an amilCP-expressing reporter operon. AmilCP belongs to a group of GFP-like CPs. CPs are small proteins that form their chromophore without needing cofactors or substrates other than oxygen. In contrast to fluorescent proteins, CPs absorb visible light to exhibit visible colours under ambient light [49]. The list of CP applications is growing. CPs such as amilCP are used as markers in molecular cloning procedures, similar to the LacZ alpha peptide [50], or in duplication-insertion recombineering of *E. coli* and *Salmonella enterica* [51]. Compared with fluorescent reporters, amilCP-producing *V. cholerae* strains are detected by colour in an instrument-free manner. Unlike LacZ, the selection of amilCP-coloured colonies does not require exogenously added substrates and is not limited by background activity from endogenous enzymes [52]. Compared with auxotrophic markers, there is no need to incubate amilCP-expressing cells on relatively expensive selective media. Notably, the amilCP reporter is safer than antibiotic resistance markers because it has no potential to be allergic to humans [53] and represents no threat to possible lateral spread in the environment.

The plasmid instability revealed by colony sectoring of *V. cholerae* (Figure 4c) was phenotypically highly similar to other cases of genetic instability [54,55,56]. Genetic instability can lead to the loss of genetic changes in engineered vaccine strains. Coupling genome modifications, e.g., the insertion of a gene for immuno-stimulating protein, in one operon with amilCP allows for rapid and visible detection of the loss of these modifications. Therefore, the amilCP-based reporter system provides a fast and straightforward way to ensure the quality of genetically engineered vaccine strains during their maintenance.

The limitation of the amilCP reporter system is that it requires a high level of amilCP expression. Therefore, strong promoters should be used to express the amilCP gene. On the other hand, visible amilCP colouration also ensures a high expression level of a heterologous protein encoded in the operon with amilCP. Therefore, the amilCP reporter system facilitates the construction of a superproducer of the immuno-stimulating protein. Additionally, the requirement of a homologous recombination limits the application of our strategy to bacterial species with a high homologous recombination level, e.g., genius *Vibrio* and *Neisseria*.

Finally, the fourth step of the strategy is the genome integration of the reporter operon expressing an immuno-stimulating protein with a simultaneous deletion of the *recA* locus. The *recA* deletion further increases the relatively high safety of the chosen candidate vaccine strains. The *recA* deficiency interferes with the acquisition of toxigenic prophages via lateral transfer [57]. Moreover, *recA* inactivation impairs homologous recombination and contributes to high genetic stability, as evidenced by cases of vaccine strains, e.g., BCG1 [58], Salmonella [59], and influenza A [60]. Significantly, simultaneous *recA* deletion by integrating an expression cassette for immunogenic proteins shortens the time for creating a genetically stable candidate vaccine strain. However, *recA* disruption abolishes further genome engineering by homologous recombination. Therefore, *recA* disruption should be performed at the final stage of vaccine strain development to lock the state of its modified genome.

The rapid development and implementation of genetically modified vaccines is an effective way to control pandemics. This is exemplified by the rapid development of the Sputnik V vaccine against SARS-CoV-2 [61,62], which helped control the COVID-19 pandemic in the Republic of San Marino. Our strategy can be used for the rapid development of vaccine strains against not only *V. cholera* but also other human bacterial pathogens to combat spreading multidrug-resistant strains with high pandemic potential.

## 4. Materials and Methods

### 4.1. Bacterial Strains

Two nontoxigenic *V. cholerae* strains were obtained from the state collection of pathogenic microorganisms and cell cultures “GKPM-Obolensk” of the State Research Center for Applied Microbiology and Biotechnology: *V. cholerae* O1 El Tor Ogawa P-19431 (cat. no. B-7504, then strain 31) and *V. cholerae* non O1/O139 P-9741 (cat. no. B-5948, then strain 41). GKPM-Obolensk also provided genomic DNA for the toxigenic strain P-19241. *Escherichia coli* TOP10 (*mcrA*, Δ(*mrr-hsd*RMS-*mcrBC*), Phi80*lacZ(del)M15*, Δ*lacX74*, *deoR*, *recA1*, *araD139*, Δ(*ara-leu*)7697, *galU*, *galK*, *rpsL(SmR)*, *endA1*, *nupG*) was purchased from Thermo Fisher Scientific (Waltham, MA, USA).

### 4.2. MALDI-TOF Mass Spectrometry

Biological samples of the strains were analysed following the protocols developed by Bruker Daltonics (Germany) (https://www.bruker.com/en/products-and-solutions/mass-spectrometry/maldi-tof/ultraflextreme.html, accessed 25 Ocotober 2021). A culture of cells in the exponential growth phase was resuspended in 70% ethanol and pelleted by centrifugation at maximum speed. The cell sediment was dried and mixed with an equal volume of 70% formic acid. The suspension was mixed with an equal volume of acetonitrile and precipitated by centrifugation at maximum speed. The supernatant containing the extracted proteins was mixed with a matrix (α-cyano-4-hydroxycinnamic acid (6 mg/mL) in acetonitrile/water/trifluoroacetic acid solution (50:47.5:2.5 *v*/*v*/*v*)), dried and crystallized on a stainless-steel substrate (Bruker Daltonics, Germany). The analysis was performed on a MALDI Biotyper ultrafleXtreme mass spectrometer (Bruker Daltonics, Germany). Raw spectra were recorded in a linear positive mode at a laser frequency of 20 Hz in the mass range from 2 to 20 kDa. Smoothing, normalization, baseline subtraction, and peak selection were performed automatically using the Flex Control software (v.3.4, build 135). The spectral data obtained using the MALDI Biotyper Compass Explorer 4.1 software were automatically compared with the reference spectral database for the collection of microorganisms supplied with the mass spectrometer. Spectra were compared using peak position, peak intensity distributions, and peak frequencies based on a built-in pattern recognition algorithm. The search results were expressed as the logarithm of the values-score values (SV). The taxonomic affiliation of the microorganisms was determined based on the SV: SV ≥ 2.3 that corresponds to reliable species identification; SV less than 2.299 but more than 2000 indicates reliable identification of the genus and likely correct identification of the species; SV between 1.7 and 1.999 is considered to indicate likely correct genus identification; and SV less than 1.7 indicates unreliable identification.

### 4.3. Sequencing of the Genomes of V. cholerae Strains

Cultures of *V. cholerae* strains grown to the stationary phase were used for genomic DNA purification. Genomic DNA was purified with a phenol:chloroform:isoamyl alcohol mixture (25:24:1 *v*/*v*/*v*) followed by RNase A treatment. Sequencing libraries were prepared using the commercial NEBNext^®^ Fast DNA Fragmentation and Library Prep Set for Ion Torrent™ (New England Biolabs, MA, USA), followed by sequencing on the Ion S5XL platform (Thermo Scientific, MA, USA). As a result of sequencing, a total of 5,904,688 (average read length of 296 nt) and 5,351,031 (average read length of 303 nt) reads were obtained for strain 31 and strain 41, respectively. Quality control and filtering of the initial reads were performed using the FaQCs program [63]. The reads were assembled into contigs using the SPAdes program [64] with the following k-mer values: 21, 33, 55, 77, 99, and 127. In the case of strain 31, 183 contigs were assembled with N50 = 188,915, an average contig length of 21333.8 nt, a maximum contig length of 362,317 nt, a minimum contig length of 202 nt, and a total contig length of 3,904,085 nt. In the case of strain 41, 173 contigs were assembled with N50 = 182,634, an average contig length of 23473.2 nt, a maximum contig length of 504,637 nt, a minimum contig length of 205 nt, and a total contig length of 4,060,870 nt.

### 4.4. Bioinformatics Analysis of V. cholerae Genome Sequences

The Microbial Genomes Atlas web server [22] searched for the most similar genome and a 16S RNA analysis. BLAST (https://blast.ncbi.nlm.nih.gov/Blast.cgi, accessed 4 February 2019) with default parameters was used to search for genes in our strains’ genome sequences. The sequences of the genes of interest of the reference strains were retrieved from the Kyoto Encyclopedia of Genes and Genomes (KEGG) Genome Database (https://www.genome.jp/kegg/genome.html, accessed 4 February 2019). Using Notepad ++ (https://notepad-plus-plus.org/, accessed 4 February 2019), FASTA files were generated for a subsequent search in a locally installed version of BLAST 2.10.0 with default parameters. Genes of CRISPR/Cas systems were detected using CRISPRminer (http://www.microbiome-bigdata.com/CRISPRminer/, accessed 5 March 2019) with default parameters. Toxin genes were searched for in the draft genomes using the Abricate (Abricate, GitHub https://github.com/tseemann/abricate, accessed 10 December 2018) and SRST2 [65] programs with the Virulence Factor Database (VFDB) [66]. Genes providing antibiotic resistance (AMR) were searched for using both reads and assembled contigs with the following programs: Resfinder (which uses the Resfinder database) [67], RGI (which uses the CARD database) [68], and AmrFinder (which uses the NCBI database) [69]. A multilocus sequence analysis was performed using the MLST 2.0 server [70].

### 4.5. Gibson Assembly

Purified PCR fragments of inserts and linearized plasmids or their amplified backbones were used in the assembly reaction using the enzymes and reaction conditions as described previously [71].

### 4.6. Transformation of Plasmid DNA by Electroporation

The *V. cholerae* strains were transformed by electroporation as described in [25] with modification. Briefly, *V. cholerae* cells from 2 mL overnight cultures were pelleted by centrifugation, resuspended, and then pelleted three times in 1 mL of a cold solution of 2 mM CaCl_2_ on ice. The supernatant was removed, and the bacterial pellet was resuspended in 40 μL of 2 mM CaCl2 on ice. Then, 1 μg of plasmid DNA was added in a volume of 1 μL to the bacterial suspension, mixed, and transferred to a 0.2 cm chilled Gene Pulser electroporation cuvette (Bio-Rad). Electroporation was performed on a Gene Pulser Xcell device (Bio-Rad). The pulse settings were as follows: pulse type: exponential, C (μF) = 25, PC (ohm) = 200, and V = 2500. Immediately after electroporation, the mixtures were suspended in 2 mL of LB broth incubated at 37 °C for 30 min with shaking at 200 rpm, plated on Petri dishes with appropriate media, and grown at RT for 12–24 h.

### 4.7. Construction of Plasmids

#### 4.7.1. pTZ57R-amilCP

The pTZ57R backbone (2746 bp) was amplified with the primers M13Fas and M13Ras (Table S1). The ORF of amilCP (666 bp) was amplified from the PGR-Blue plasmid (Addgene No. 68374) with the primers VC-01 and VC-02. The amplified fragments were purified, Gibson assembled, and transformed into *E. coli* through electroporation. *E. coli* colonies with violet colouration were used to purify the plasmid. The correctness of the plasmid assembly was checked by PCR and a restriction analysis followed by partial Sanger sequencing.

#### 4.7.2. pCI-amilCP

The pEGFP-C1 (Clontech, Mountain View, CA, USA) backbone (2690 bp) was amplified using the primers VC-03 and VC-04. The left flank (3069 bp) upstream of the *V. cholerae* β-galactosidase ORF was amplified from the genomic DNA of the toxigenic *V. cholerae* strain P-19241 using the primers VC-05 and VC-06. The ORF of amilCP (666 bp) was amplified from the PGR-Blue plasmid (Addgene No. 68374), with the primers VC-07 and VC-08. The right flank (3137 bp), the full-length ORF of *V. cholerae* β-galactosidase, was amplified from the *V. cholerae* strain P-19241 genomic DNA with the primers VC-09 and VC-10. The amplified fragments were purified, Gibson assembled, and transformed into *E. coli* through electroporation. *E. coli* colonies with violet colouration were used to purify the plasmid. The correctness of the plasmid assembly was checked by PCR a and restriction analysis followed by partial Sanger sequencing.

#### 4.7.3. pALAL

The pEGFP-C1 backbone (2690 bp) was amplified using the primers VC-03 and VC-04. The right (3137 bp) and left (3069 bp) flanks were amplified from the pCI-amilCP plasmid using the primer pairs VC-09/VC-10 and VC-05/VC-11, respectively. The β-lactamase ORF (861 bp) was amplified from the pTZ57R vector with the primers VC-12 and VC-13. The lacY ORF (1254 bp) was amplified from the *E. coli* genomic DNA using the primers VC-14 and VC-15. The amilCP ORF (666 bp) was amplified from the PGR-Blue plasmid (Addgene No. 68374) with the primers VC-16 and VC-08. The amplified fragments were purified, Gibson assembled, and transformed into *E. coli* through electroporation. *E. coli* colonies were grown on agar plates supplemented with ampicillin and kanamycin, and those with violet colouration were used to purify the plasmid. The correctness of the plasmid assembly was checked by PCR and a restriction analysis followed by partial Sanger sequencing.

#### 4.7.4. pCI-RACR-0.5

The pEGFP-C1 backbone (2690 bp) was amplified using the primers VC-21 and VC-22. The left flank (640 bp) upstream of the *V. cholerae recA* ORF was amplified from the genomic DNA of *V. cholerae* strain 31 using the primers VC-23 and VC-24. The amilCP ORF (666 bp) was amplified from the PGR-Blue plasmid (Addgene No. 68374) with the primers VC-25 and VC-26. The chloramphenicol acetyltransferase gene (660 bp) was amplified from the plasmid pCas9 (Addgene No. 42876) using the primers VC-27 and VC-28. The right flank (591 bp) downstream of the *V. cholerae recA* ORF was amplified from the genomic DNA of *V. cholerae* strain 31 using the primers VC-29 and VC-30. The amplified fragments were purified, Gibson assembled, and transformed into *E. coli* through electroporation. *E. coli* colonies were grown on agar plates supplemented with chloramphenicol (Cm) and kanamycin, and those with violet colouration were used to purify the plasmid. The correctness of the plasmid assembly was checked by PCR and a restriction analysis followed by partial Sanger sequencing.

#### 4.7.5. pCI-RACR-3.0

The pEGFP-C1 backbone (2690 bp) was amplified using the primers VC-31 and VC-32. The left flank (3141 bp) upstream of the *V. cholerae recA* ORF was amplified from the genomic DNA of *V. cholerae* strain 31 using the primers VC-33 and VC-24. The amilCP ORF (666 bp) was amplified from the PGR-Blue plasmid (Addgene No. 68374) with the primers VC-25 and VC-34. The chloramphenicol acetyltransferase gene (660 bp) was amplified from the plasmid pCas9 (Addgene No. 42876) using the primers VC-35 and VC-28. The right flank (3094 bp) downstream of the *V. cholerae recA* ORF was amplified from the genomic DNA of *V. cholerae* strain 31 using the primers VC-29 and VC-36. The amplified fragments were purified, Gibson assembled, and transformed into *E. coli* through electroporation. *E. coli* colonies were grown on agar plates supplemented with Cm and kanamycin, and those with violet colouration were used to purify the plasmid. The correctness of the plasmid assembly was checked by PCR and a restriction analysis followed by partial Sanger sequencing.

#### 4.7.6. pCI-RCCACR-3.0

The pEGFP-C1 backbone (2690 bp) was amplified using the primers VC-31 and VC-32. The left flank (3048 bp) upstream of the *V. cholerae recA* ORF was amplified from the genomic DNA of *V. cholerae* strain 31 using the primers VC-33 and VC-37. The DNA fragments of the promoter region of the cholera toxin operon (211 bp) and *ctxB* ORF (375 bp) were amplified from the genomic DNA of the *V. cholerae* strain P-19241 using the primer pairs VC-38/VC-39 and VC-40/VC-41, respectively. The synthetic operon containing genes encoding amilCP and chloramphenicol acetyltransferase (1342 bp) was amplified from the pCI-RACR-3.0 plasmid using the primers VC-42 and VC-28. The right flank (3094 bp) downstream of the *V. cholerae recA* ORF was amplified from the genomic DNA of *V. cholerae* strain 31 using the primers VC-29 and VC-36. The amplified fragments were purified, Gibson assembled, and transformed into *E. coli* through electroporation. *E. coli* colonies were grown on agar plates supplemented with Cm and kanamycin, and those with violet colouration were used to purify the plasmid. The correctness of the plasmid assembly was checked by PCR and a restriction analysis followed by partial Sanger sequencing. The plasmids used in this work are described in Table S2.

### 4.8. Measurement of Cholera Toxin β-Subunit Production

The choleral toxin, β-subunit, production was estimated using GM1-ELISA as described in [72]. The calibration curve was constructed using purified cholera toxin β-subunit (Sigma-Aldrich, St. Louis, MO, USA) prepared as a 3-fold dilution in phosphate-buffered saline (PBS). The toxin was detected using a primary rabbit anti-CTX antibody (1:10,000, Sigma-Aldrich, St. Louis, MO, USA) and a secondary peroxidase-conjugated anti-rabbit antibody (1:20,000, Jackson ImmunoResearch Laboratories, West Grove, PA, USA). Toxin concentrations were measured in cell lysates and the culture media of the *V.* *cholerae* strains using a calibration curve.

## Data Availability

The data presented in this study are available on reasonable request from the corresponding author.

## Supplementary Materials

The following are available online at https://www.mdpi.com/article/10.3390/ijms222111657/s1.

## Abbreviations

BHI	brain heart infusion
Cm	chloramphenicol
CP	chromoprotein
CRISPR	clustered regularly interspaced short palindromic repeats
ELISA	enzyme-linked immunosorbent assay
KEGG	Kyoto Encyclopedia of Genes and Genomes
MALDI-TOF	matrix-assisted laser desorption/ionization time of flight
MLST	multi-locus sequence typing
OCV	oral cholera vaccine
ONPG	ortho-nitrophenyl-β-galactoside
PBS	phosphate-buffered saline
SV	score value
VFDB	Virulence Factor Database
X-Gal	5-bromo-4-chloro-3-indoyl-beta-D-galactopyranoside
