# Supplementary material for: A Strategy for the Rapid Development of a Safe Vibrio cholerae Candidate Vaccine Strain
Source: Int J Mol Sci. 2021 Oct 28;22(21):11657. doi: 10.3390/ijms222111657 (PMC8583953; doi:10.3390/ijms222111657)
Supplement: Supplementary file 1 [file ijms-22-11657-s001.zip › ijms-1425015-supplementary.pdf]

Supplementary Methods

1.1. Cultivation of strains of V. cholerae

All work with *V. cholerae* cultures was carried out in a boxing class 2 biological protection Thermo Herasafe KS (Thermo Scientific, Waltham, MA, USA). Strains of *V. cholerae* were cultured in LB (lysogeny broth) medium containing tryptone (10.0 g/L), yeast extract (5.0 g/L), and NaCl (5.0 g/L) and prepared by following the instructions described in the Gene Pulser Xcell electroporation system instruction manual (Bio-Rad, CA, USA). Agarized LB medium was prepared by adding agar to the LB medium (15 g/L) and subsequently autoclaving (at 121 °C for 30 minutes). When preparing agar medium with antibiotics, the medium was melted and cooled to 55–60 °C, after which the required amount of a solution of the necessary antibiotic was added. The medium was poured into 12-15 ml disposable Petri dishes with a diameter of 100 mm (ZAO Perint, Russia). The medium was allowed to solidify and used after drying the condensate. Bacterial cultures were incubated in a dry-air thermostat TC 1/80 (Smolensk SKTB SPU, Russia). For long-term storage of laboratory strains, cryopreservation was performed in Cryoinstant cryovials (Deltalab, Spain), which were stored at -70 °C.

1.2. Preparation of M9 minimal medium

M9 medium was prepared according to the instructions provided at http://subtiwiki.uni-goettingen.de/wiki/index.php/M9_minimal_medium. One litre of 5x M9 stock solution was prepared by mixing 42.5 g of $Na_2HPO4 \cdot 2H_2O$, 15 g of KH_2PO4, 5.0 g of NH_4Cl, and 2.5 g of NaCl. Using 4 M NaOH, the pH of the solution was adjusted to 7. The solution was autoclaved at 121 °C for 20 minutes and stored at room temperature. One litre of a 100x stock solution of trace elements was prepared by mixing 100 mg of $MnCl_2$ • 4H2O, 170 mg of $ZnCl_2$, 43 mg of $CuCl_2$ • 2H2O, 60 mg of $CoCl_2$ • $6H_2O$, and 60 mg of Na_2MoO_4 • $2H_2O$. Separately, solutions of 100 mM $CaCl_2$ • $2H_2O$ (1.47 g per 100 ml) and 1 M $MgSO_4$ • $7H_2O$ (24.6 g per 1 L) were prepared, and these solutions were autoclaved and stored at room temperature. A solution of 50 mM $FeCl_3 \cdot 6H_2O$ in 100 mM citric acid was prepared by dissolving 1.35 g of iron chloride and 2.10 g of citric acid monohydrate in 100 ml of deionized water. The solution was sterilized by filtration and stored in the dark at 4 °C. One litre of 50% and 20% (mass/volume) glucose solutions was prepared by dissolving 500 and 200 g of glucose in deionized water in a warm water bath; the solutions were autoclaved at 121 °C for 20 minutes and stored at room temperature. One litre of a 13.4% solution of L-malate was prepared by dissolving 134 g of L-malate in deionized water and adding 470 ml of 4 M NaOH to adjust the pH to 7. The solution was sterilized by filtration. One litre of M9 medium was prepared by mixing the stock solutions in the following order: 200 ml of 5x stock solution of M9, 1 ml of 100 mM $CaCl_2$, 10 ml of 100x stock solution of trace elements, 1 ml of 1 M $MgSO_4$, 1 ml of 50 mM $FeCl_3$/100 mM citric acid, and 6 ml of 50% glucose (or 15 ml of 20% glucose or 15 ml of 20% DL-malate) to prepare a medium with 0.3% carbon source or 10 ml of 50% glucose (or 25 ml of 20% glucose or 25 ml of 20% DL malate) to prepare a medium with 0.5% carbon source. The solution volume was brought up to 1 litre with deionized water.

1.3. Microscopy of V. cholerae strains

The study of the bacterial cultures' micromorphological and tinctorial properties was carried out by microscopy of stained smears. The reagents were prepared and Gram-stained using the Gram Stain Kit (BD BBL, USA) following the manufacturer's instructions. Light microscopy of the stained preparations was carried out on an MX-100 microscope (Microoptix, Austria) in an immersion system at a magnification of 1000x.

1.4. Analysis of the ability of V. cholerae strains to ferment glucose, lactose, and sucrose

The ability of the strains to ferment glucose, lactose, and sucrose was analysed on iron TSI agar (Conda Frondisa, Spain) (https://www.peertechzpublications.com/special-issue-articles/IJVSR-S1-110.pdf). Beveled test tubes containing medium were prepared;

the length of the bevel was 1/3 and that of the column was 2/3 the height of the medium in the test tube. Inoculation was carried out with a bacterial needle, first injecting cells into the bevel and then, with the same needle, seeding the surface of the bevel. The tubes were incubated at room temperature for one day.

1.5. Determination of β-galactosidase activity in liquid and solid media

The presence of β-galactosidase activity in the cell lysates of the strains was determined by the ability to cleave O-nitrophenyl-β-galactopyranoside (ONPG) with the release of O-nitrophenol, which leads to the appearance of a yellow colour in the solution (https://www.kbmk.kirov.ru/library/7/doc/1/index1.4.htm). A 0.75 M stock solution of ONPG (Sigma-Aldrich, St. Louis, MO, USA) was prepared. A working aqueous solution diluted 100-fold was prepared. Bacterial cultures were added to 1 ml of the ONPG working solution (1 tank loop), and the colour change of the solution was observed. The presence of β-galactosidase activity and galactoside transport systems inside the cells was determined using 5-bromo-4-chloro-3-indoyl-beta-D-galactopyranoside (X-Gal). A stock 1000-fold solution of X-Gal (Thermo Scientific, USA) in DMSO (20 mg/ml) was prepared. The X-Gal solution was introduced into molten LB agar at a final concentration of 20 μg/ml, and the medium was poured into Petri dishes. The agar was allowed to cool, and cells strained from the liquid cultures were streaked onto the surface. The Petri dishes were incubated at room temperature, and the colour change of the colonies was observed. If X-Gal is transported into cells, it is cleaved by β-galactosidase to form 5-bromo-4-chloro-3-hydroxy indole, which, when oxidized, forms an insoluble dye, 5,5'-dibromo-4,4'-dichloro-indigo, which determines colony colouration.

1.6. Determination of the haemolytic activity of V. cholerae strains

The haemolytic properties of the strains were studied on Columbia agar with defibrinated blood (manufactured by TsFGS LLC, Russia). The composition of the medium was as follows: peptones (23 g/l), starch (1 g/l), NaCl (5 g/l), agar (11 g/l), and defibrated lamb blood (5%, added to the remaining components after autoclaving them and cooling to a temperature of 40-45 °C). The cooked agar was melted and poured into Petri dishes. Single colonies of the strains were inoculated on the agar surface and incubated at room temperature for 24 h.

1.7. Sanger sequencing of DNA fragments

The amplified DNA fragments were separated by 1.2% agarose gel electrophoresis and purified using the GeneJET™ PCR Clean Up Kit (Thermo Scientific, No. K0701) according to the manufacturer's instructions. Purified fragments (20 ng) were evaporated together with the oligonucleotides used for amplification; only one primer was used in each tube. The sequencing reaction was carried out with a set of reagents (ABI PRISM® BigDye™ Terminator v. 3.1), then the reaction products were analysed on an automated DNA analyser (Applied Biosystems 3730).

Supplementary Tables

Table S1. Oligonucleotides used in this work.

Name	Sequence (5'->3')
M13Fas	GTCGTGACTGGGAAAACCCTGGCG
M13Ras	GTCATAGCTGTTTCCTGTGTGA
VC-01	TTGGGTAACGCCAGGGTTTTCCCAGTCACGACTTAGGCGACCACAGGTTTGC
VC-02	CAATTTCACACAGGAAACAGCTATGACCATGAGTGTGATCGCTAAACAAATG
VC-03	GCCTTCAACCCAGTCAGCTCCTTCCGAGATCTCAGGTGGCACTTTTCGGGGAA
VC-04	CCTAGTTCTTTTAAAGACTGAGTTCTGTTTAGATCTCGGAACTCCATATATGGGCTATGAACT

VC-05	AAACAGAACTCAGTCTTTAAAAGAACTAGGGCTCAAAACCGGATCCGAG-CACAAGGAGGGTGATTGAAC
VC-06	GTATTTCTCCTCTTTAATTACTACCCTCAAGCCGAGGAGTAAAGAAGTCATTC
VC-07	ACTCCTCGGCTTGAGGGTAGTAATTAAAGAGGAGAAATACTAGATGAGTGTGATCGC
VC-08	ACGGCGTACATTTTTCAACTACTCCTGTTAGGCGACCACAGGTTTGCG
VC-09	CGGAAGGAGCTGACTGGGTTGAAGGCTCTCAAGGGCATCGGTCGACTTATTGTGGTGGG-GATGACGCT
VC-10	GGTCGCCTAACAGGAGTAGTTGAAAAATGTACGCCGTAGAGCAAAGG
VC-11	CGGAAATGTTGAATACTCATCTAGTATTTCTCCTCTTTAATTACTACCCTC
VC-12	AGTAATTAAAGAGGAGAAATACTAGATGAGTATTCAACATTTCCGTGTCG
VC-13	TTTGTGTTTTTTAAATAGTACATTACTTACTCCTAGACGTTACCAATGCTTAATCAGTGAGGCA
VC-14	AGCATTGGTAACGTCTAGGAGTAAGTAATGTACTATTTAAAAAACACAAACTTTTGGATG
VC-15	TGACATATAAACCTTGTAGGTCATTTGTTTAGCGATCACAC-TCATTCTTCTAATCTCCCTTAAGCGACTTCATTCACCTG
VC-16	ATGAGTGTGATCGCTAAACAAATGACCT
VC-17	CAGAAATGAACAGGGTTGGAC
VC-18	CATCTTTTACTTTCACCAGCGT
VC-19	GATGCCAGGGTATCACTATGT
VC-20	CAGTGATTGGTTTTGACGATG
VC-21	GCTCACTCAAGGAATGCAGTGTTTTCATAGATCTCGGAACTCCATATATGGGCTATGAACTA
VC-22	GCTTCAAGCCAGTGAGAGTGATGAGTAAAGATCTCAGGTGGCACTTTTCGGGGAA
VC-23	ATGAAAACACTGCATTCCTTGAGTGAGC
VC-24	CATTTGTTTAGCGATCACACTCATTACTCTCTCCGGATAGTCACTCTCAGG
VC-25	ATGAGTGTGATCGCTAAACAAATG
VC-26	CAGTGATTTTTTTCTCCATTCTTCTAATCTCCCTTAGGCGACCACAGGTTTGCG
VC-27	GGGAGATTAGAAGAATGGAGAAAAAAATCACTGG
VC-28	CAGCCTGCCGATTACGCCCCGCCCTGCCACTC
VC-29	GAGTGGCAGGGCGGGGCGTAATCGGCAGGCTGAATACAAAGAGGT
VC-30	TTACTCATCACTCTCACTGGCTTGAAGC
VC-31	AGATCTCGGAACTCCATATATGGGCTATGAACT
VC-32	AGATCTCAGGTGGCACTTTTCGGGGAA
VC-33	CCATATATGGAGTTCCGAGATCTCCGTGAAGAAGAAGAAGCGGGCA
VC-34	TTTTCTCCATTACTTACTCCTAGACGTTAGGCGACCACAGGTT
VC-35	TAACGTCTAGGAGTAAGTAATGGAGAAAAAAATCACTGGATATACCACCG
VC-36	TTCCCCGAAAAGTGCCACCTGAGATCTATCATCTACCATGCCACGTAGCGCTT
VC-37	CAAAGGTATCGAACACCACATTTGTACAGTGTCCACCCCTGTATAG
VC-38	GTACAAATGTGGTGTTCGATACCTTTGCAGC
VC-39	AATTCATCCTTAATTCTCCTTTGTTTAACAGAAAAATAATTGATCAAAACA
VC-40	ACAAAGGAGAATTAAGGATGAATTATGATTAAATTAAAATTTGGTGTTTTTTTTACAG
VC-41	ATTCTTCTAATCTCCCTTAATTTGCCATACTAATTGCGGCA
VC-42	GGCAAATTAAGGGAGATTAGAAGAATGAGTGTGATCGCTAAAC
VC-43	AGTTAGGCATCAAGGTCGCT
VC-44	ATTTGTACAGTGTCCACCCCTGTATAG
VC-45	TCGGCAGGCTGAATACAAAGAGGT
VC-46	ACCACCCTTACCACCGACTTGTAGTGC
VC-47	CCTTCGACCTCAAAGTAGTGT
VC-48	CAACATAGTGATACCCTGGCAT

Table S2. Plasmids used in this work.

Name	Description
pTZ57R	Commercial vector (Thermo Scientific, USA)
pTZ57R-amilCP	pTZ57R derivative encoding amilCP chromoprotein
pCI	Commercial vector (Promega, USA)
pCI-amilCP	pCI derivative for amilCP integration into the *V. cholerae* lacZ locus
pALAL	pCI derivative carrying the synthetic reporter operon AmpR-lacY-amilCP integration into the *V. cholerae* lacZ locus
pCI-RACR-0.5	pCI derivative carrying the synthetic reporter operon amilCP-CmR under the control of the *V. cholerae* recA promoter
pCI-RACR-3.0	pCI derivative carrying the synthetic reporter operon amilCP-CmR integrated into the *V. cholerae* recA locus
pCI-RCCACR-3.0	pCI derivative carrying the synthetic reporter operon ctxB-amilCP-CmR under the control of the CTX promoter for integration into the *V. cholerae* recA locus

Table S3. Search results for prophages and accessory toxins in the draft genome sequences of *V. cholerae* strains.

Accessory toxin	Strain 41	Strain 31
CTXα	With frameshift mutations	No
CTXβ	No	No
hlyA	**Yes**	With frameshifting indels
hlyB	**Yes**	**Yes**
rtxA	With frameshifting indels	With frameshifting indels
rtxB	**Yes**	**Yes**
rtxC	**Yes**	**Yes**
hap	**Yes**	**Yes**
zot	No	No
Ace	No	No
Cholix toxin	No	No
Heat stable toxin (sto, ST) and context	No	No
acfA	No	No
acfB	No	No
acfC	No	No
acfD	No	No
tcpA	No	No
TLC prophage	No	No

CTX prophage	No	No
RS1 prophage	No	No

Table S4. Results of the antibiotic resistance test.

**Antibiotic**	**Concentration (µg/ml)**	**Strain 31**	**Strain 41**
Ampicillin	0	Abundant growth (++++)	Abundant growth (++++)
	25	Single colonies (+)	Abundant growth, small colonies (++++)
	50	No colonies	Moderate growth, small colonies (+++)
Kanamycin	25	Abundant growth (++++)	Abundant growth (++++)
	50	Abundant growth (++++)	Abundant growth (++++)
Chloramphenicol	15	Single colonies (+)	Single colonies (+)
	25	No colonies	No colonies

Supplementary Figures

Strain 31

Analyte Name:	V.c.31K
Analyte Description:	D:\Data\BIOTYPER_LAB\2019\10\07\V.c.31K\0_M17\1\1SLin
Analyte ID:	435ce8d6-87c2-4741-a260-9cf5639db0ae
Analyte Creation Date/Time:	
Applied MSP Library(ies):	
Applied Taxonomy Tree:	Bruker Taxonomy

Rank (Quality)	Matched Pattern	Score Value	NCBI Identifier
1 (++)	Vibrio albensis LMG 4406T HAM	2.01	140100
2 (-)	Vibrio mimicus LMG 7896T HAM	1.41	674
3 (-)	Vibrio nigripulchritudo LMG 3896T HAM	1.35	28173
4 (-)	Enterobacter cloacae MB11506_1 CHB	1.32	550
5 (-)	Delftia acidovorans DSM 39T HAM	1.31	80866

Strain 41

Analyte Name:	V,c 41K
Analyte Description:	D:\Data\BIOTYPER_LAB\2019\10\07\V,c 41K\0_M15\1\1SLin
Analyte ID:	0a099760-757d-49e3-8158-07598e08e6ad
Analyte Creation Date/Time:	
Applied MSP Library(ies):	
Applied Taxonomy Tree:	Bruker Taxonomy

Rank (Quality)	Matched Pattern	Score Value	NCBI Identifier
1 (+)	Vibrio albensis LMG 4406T HAM	1.88	140100
2 (-)	Vibrio mimicus LMG 7896T HAM	1.45	674
3 (-)	Arthrobacter woluwensis DSM 10495T DSM	1.39	156980
4 (-)	Enterobacter cloacae DSM 6234 DSM	1.33	550
5 (-)	Vibrio parahaemolyticus DSM 15477 DSM	1.32	670

Figure S1. MALDI-TOF results for identification of *V. cholerae* strains.

Strain 31

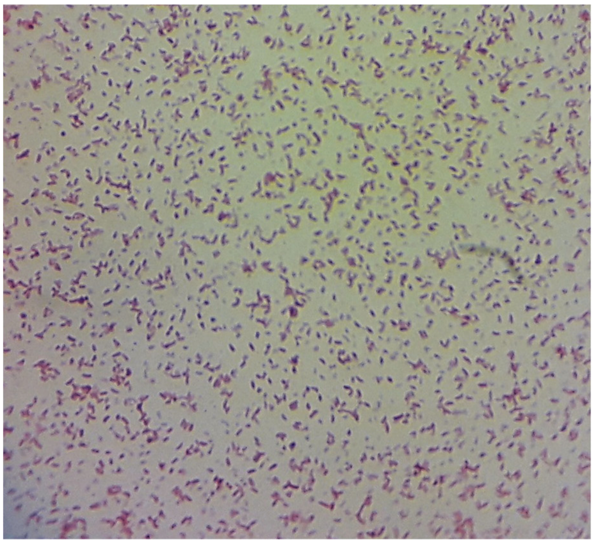

Strain 41

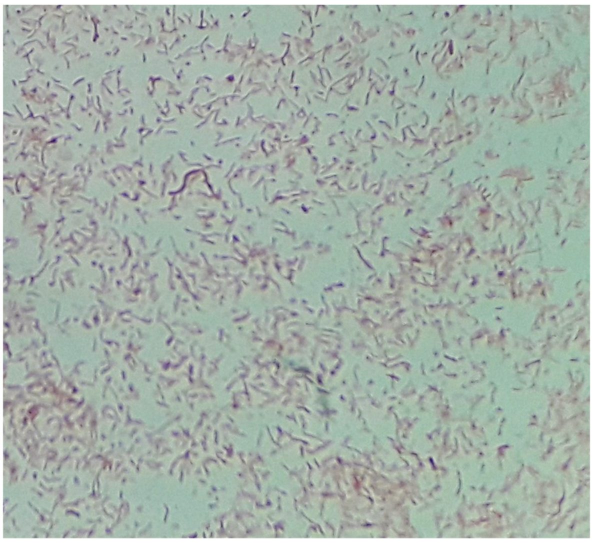

Figure S2. Microscopy of *V. cholerae* strains after Gram staining. Cells of *V. cholerae* strain 31 are gram-negative, small, rod-shaped, ovoid, and located singly or in pairs. Cells of *V. cholerae* strain 41 are gram-negative, long, thin, straight, and slightly curved sticks that are mostly single and sometimes form S-like structures.

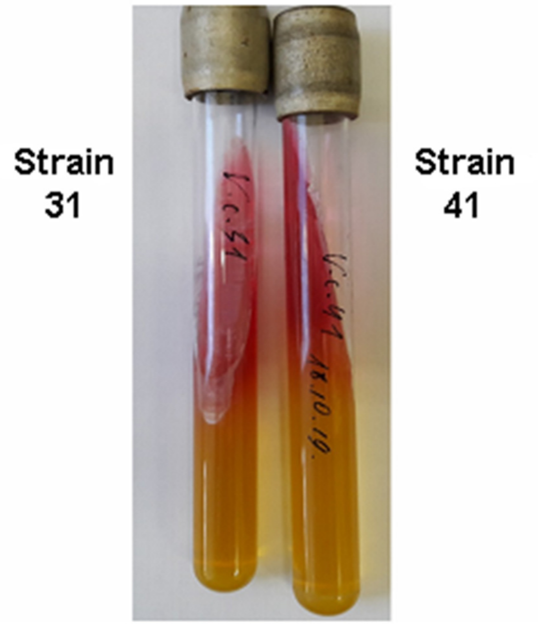

Figure S3. Biochemical test results for the ability of *V. cholerae* strains to ferment lactose, glucose, and sucrose. Both strains are capable of fermenting glucose, as evidenced by the yellow staining of the medium column. In contrast, the slope of the medium remained red for both strains, which indicates their inability to ferment lactose and sucrose.

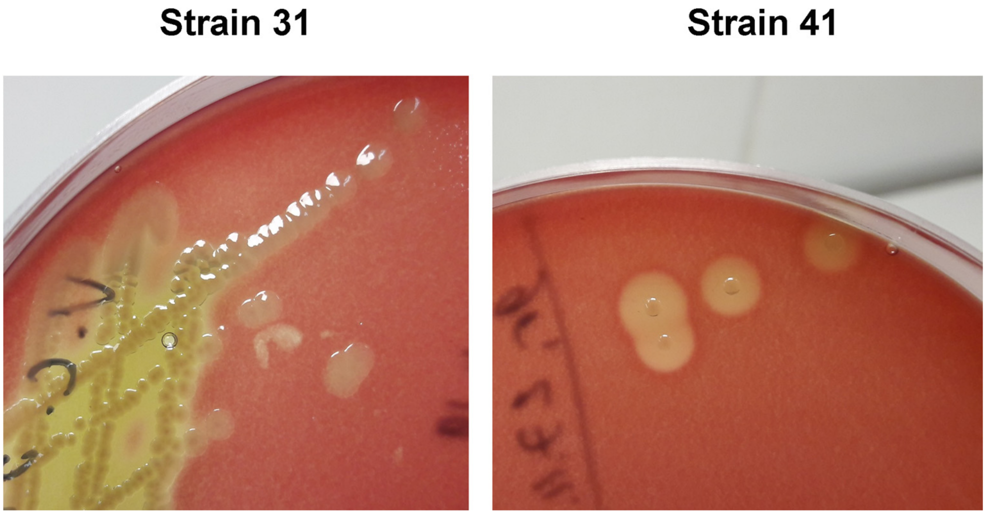

Figure S4. Formation of zones of haemolysis by the *V. cholerae* strains on blood agar. Colonies were cultured on Columbia blood agar for 24 h. Strain 31 formed colourless translucent smooth colonies with even edges; the colonies were 2–3 mm in diameter. The zone of haemolysis did not exceed the diameter of the colony, suggesting weak haemolysis (α-type). Strain 41 formed transparent drop-shaped smooth, shiny colonies that were 1-2 mm in diameter. In contrast to strain 31, a clear zone of complete haemolysis (β-type) was observed around single colonies of strain 41. The diameter of the haemolysis zones significantly exceeded the diameter of the colonies and was approximately 5–7 mm.

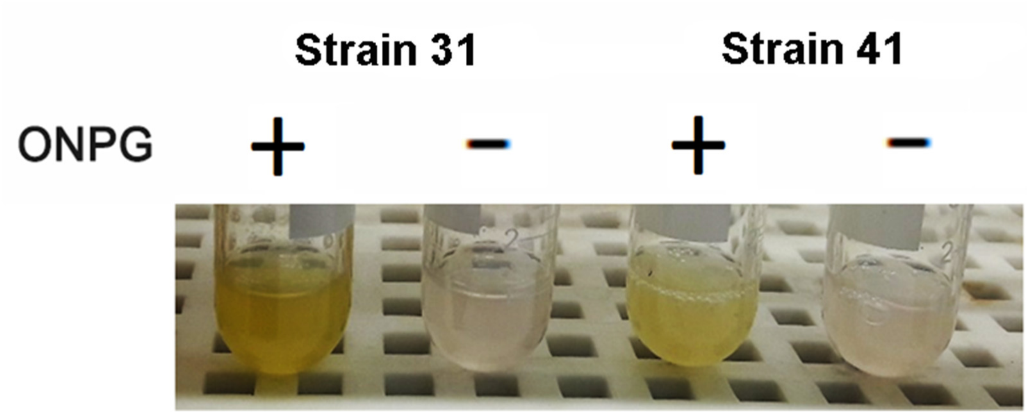

Figure S5. Colorimetric analysis of the β-galactosidase activity in cell lysates of *V. cholerae* strains. Colourless tubes: negative control without the addition of ONPG. Cell lysates were incubated with ONPG solution for 15 minutes at 37 °C. Bright yellow solutions indicate the presence of active β-galactosidase in both the studied strains.

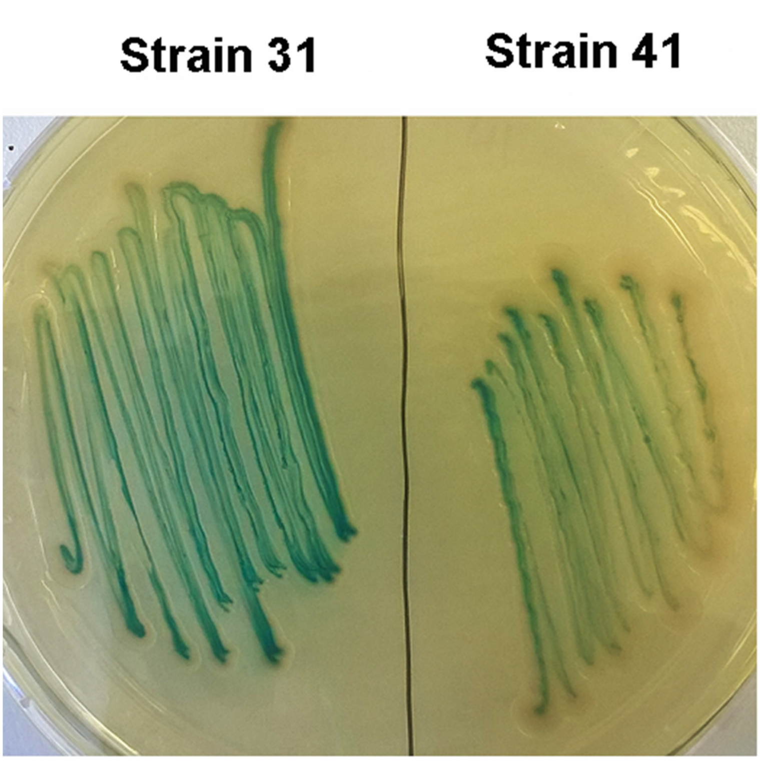

Figure S6. Conversion of the chromogenic β-galactosidase substrate 5-Bromo-4-chloro-3-indoyl-beta-D-galactopyranoside (X-Gal) by the *V. cholerae* strains. Strains were grown on agar plates with X-Gal for 48 h at room temperature. The blue colouration of bacterial colonies suggests the transport of the substrate into cells and their hydrolysis by β-galactosidase. Notably, the colonies of strain 31 developed a more intense colour.

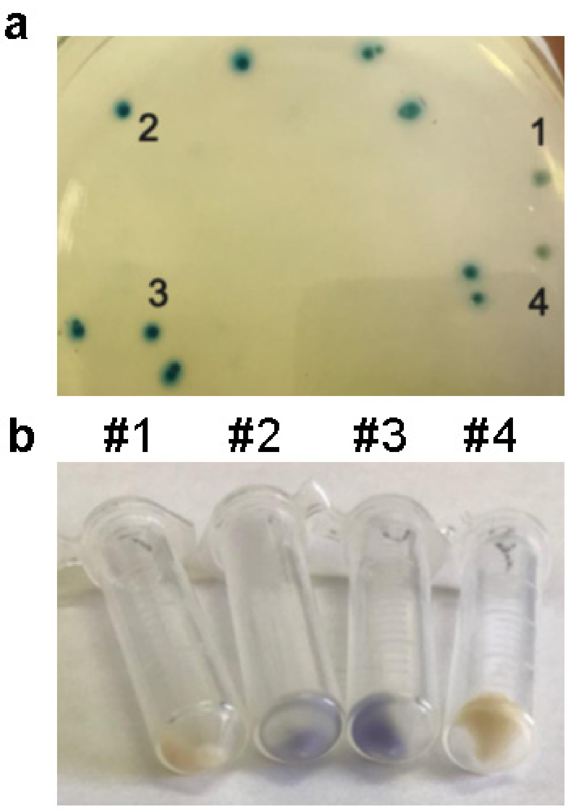

Figure S7. Phenotypes of *E. coli* cells carrying the correctly assembled pALAL plasmid. (**a**). Colony colours on the X-Gal plate supplemented with kanamycin and ampicillin. (**b**). Colour of cell pellets from selected colonies. Colonies #1 and #4 showed less intense staining than colonies 2 and 3 on X-Gal medium, which may indicate that their colour was determined only by the activity of the lacY and lacZ genes present in the *E. coli* genome. However, the more intense colouration of colonies #2 and #3 could have been provided by additional copies of lacY and lacZ genes in the assembled reporter construct. Accordingly, cell precipitates from liquid cultures grown from colonies #1 and #4 had the usual colour, while cell pellets from colonies #2 and #3 were blue due to amilCP accumulation. Therefore, colonies #2 and #3 contained correctly assembled reporter operons, which was confirmed by Sanger sequencing.

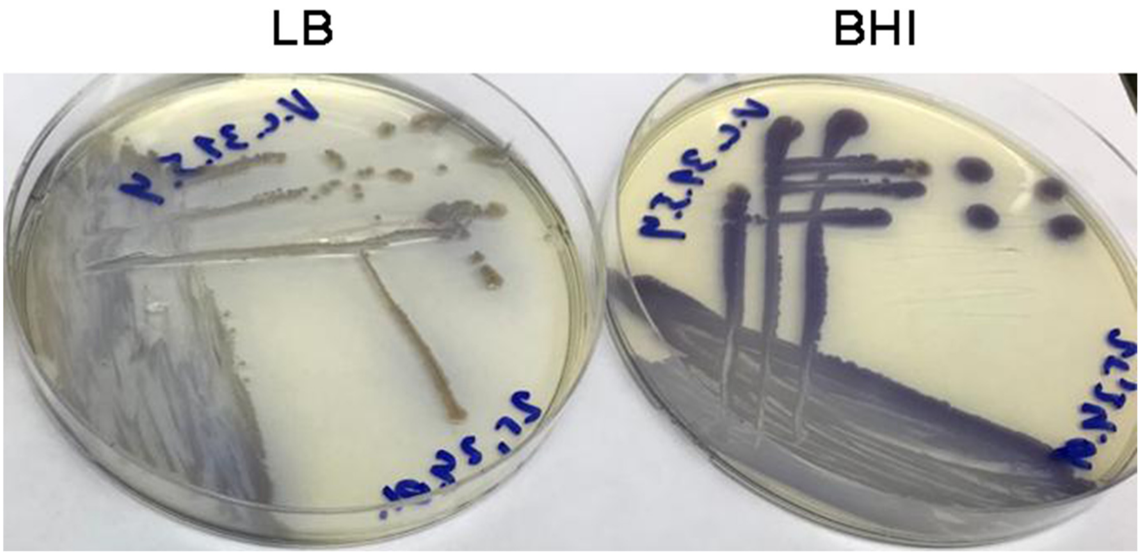

Figure S8. Optimization of culture media for increased expression of the amilCP-containing operon.

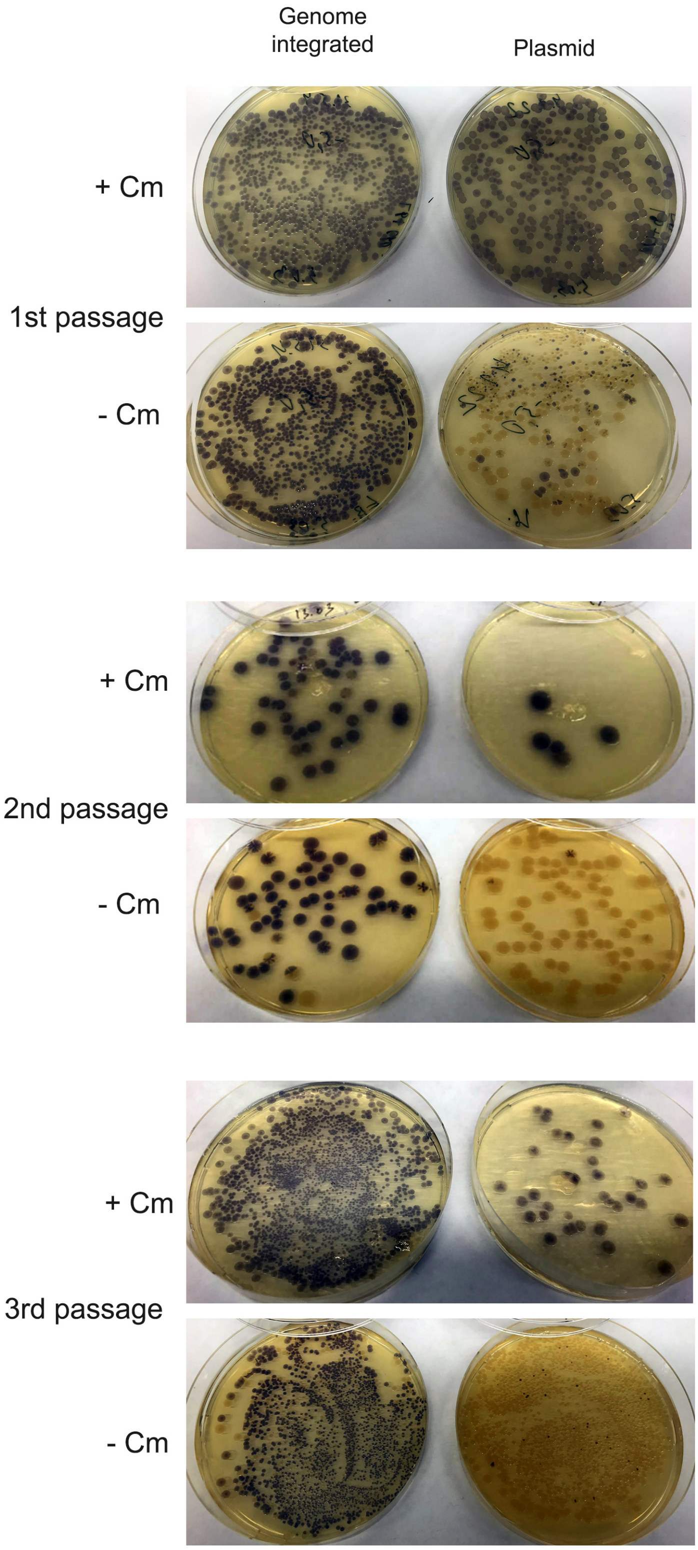

Figure S9. Representative plates for the genetic construct stability test.

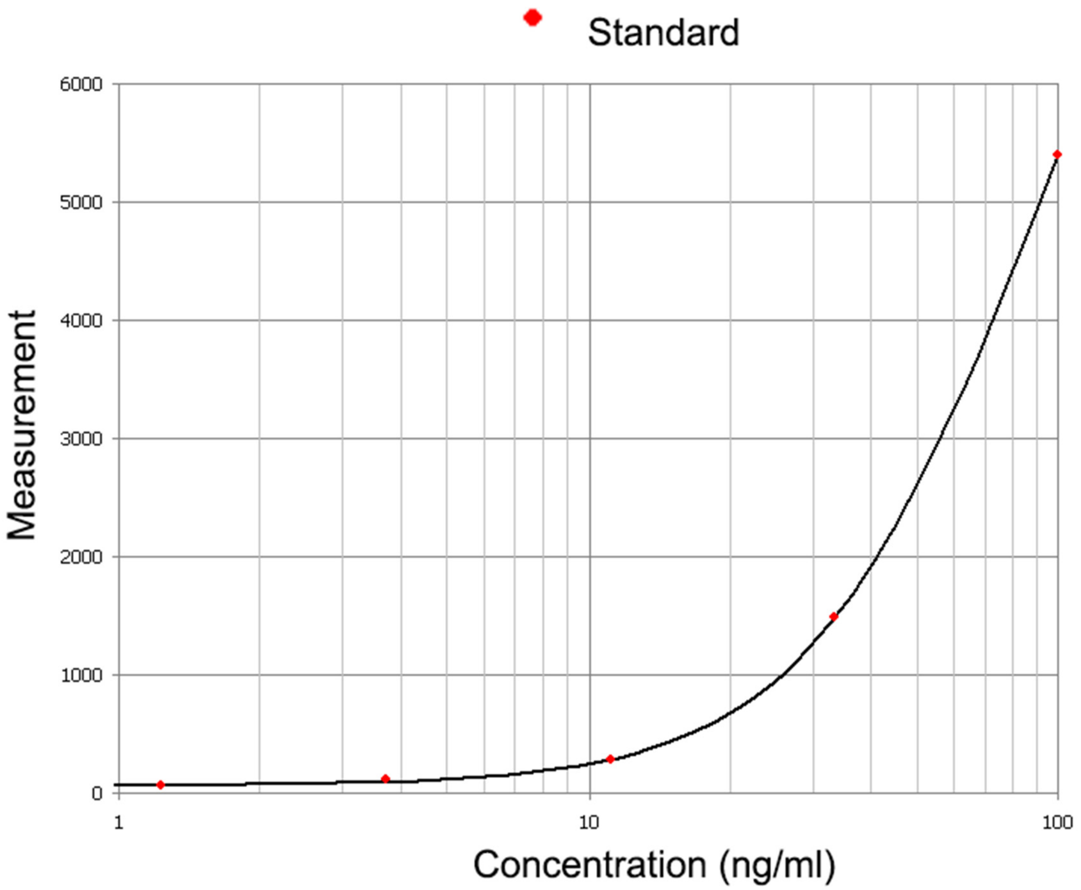

Calibrator	Well	Conc.	Raw	Backfit	Recovery %
Standard1	A1	100	5400	100	100
Standard2	B1	33,33	1480	33,34	100
Standard3	C1	11,11	282	10,95	98,59
Standard4	D1	3,704	114	4,841	130,7
Standard5	E1	1,235	59	< Curve	-
Standard6	F1	0,4115	55	< Curve	-

Figure S10. Calibration curve for quantification of β-subunit production by the rVCH-31.1 candidate vaccine strain.
